# Insecticidal toxins from black widow spider venom

**DOI:** 10.1016/j.toxicon.2006.11.021

**Published:** 2007-03-15

**Authors:** A. Rohou, J. Nield, Y.A. Ushkaryov

**Affiliations:** Division of Cell and Molecular Biology, Imperial College London, Exhibition Road, London, SW7 2AZ, UK

**Keywords:** Latroinsectotoxin, *α*-latrotoxin, 3D structure, Sequence homology, Pore formation, Receptors

## Abstract

The biological effects of *Latrodectus* spider venom are similar in animals from different phyla, but these symptoms are caused by distinct phylum-specific neurotoxins (collectively called latrotoxins) with molecular masses ranging from 110 to 140 kDa. To date, the venom has been found to contain five insecticidal toxins, termed *α*, *β*, *γ*, *δ* and *ε*-latroinsectotoxins (LITs). There is also a vertebrate-specific neurotoxin, *α*-latrotoxin (*α*-LTX), and one toxin affecting crustaceans, *α*-latrocrustatoxin (*α*-LCT). These toxins stimulate massive release of neurotransmitters from nerve terminals and act (1) by binding to specific receptors, some of which mediate an exocytotic signal, and (2) by inserting themselves into the membrane and forming ion-permeable pores. Specific receptors for LITs have yet to be identified, but all three classes of vertebrate receptors known to bind *α*-LTX are also present in insects. All LTXs whose structures have been elucidated (*α*-LIT, *δ*-LIT, *α*-LTX and *α*-LCT) are highly homologous and have a similar domain architecture, which consists of a unique N-terminal sequence and a large domain composed of 13–22 ankyrin repeats. Three-dimensional (3D) structure analysis, so far done for *α*-LTX only, has revealed its dimeric nature and an ability to form symmetrical tetramers, a feature probably common to all LTXs. Only tetramers have been observed to insert into membranes and form pores. A preliminary 3D reconstruction of a *δ*-LIT monomer demonstrates the spatial similarity of this toxin to the monomer of *α*-LTX.

## 1 Introduction

Species of the genus *Latrodectus* (Arthropoda: Chelicerata: Arachnida: Araneae: Theridiidae) are commonly known as “widow” spiders. The name of the black widow spider, *Latrodectus mactans*, is a mixture of Latin and Greek, meaning “deadly biting robber” (from *latro*—robber, bandit; *δαγκάνω*—to bite; *macto*—to slay). Indeed, its bites cause severe pain and other serious clinical symptoms in humans, making this spider medically important. However, black widow venom evolved mainly to immobilise and/or kill insects, the spider's natural prey. Toxicity against vertebrates is likely to have evolved as a means to protect the species against predation and accidental crushing. The venom has also been shown, at least under laboratory conditions, to paralyse or kill crustaceans, some of which may actually be hunted by the spider.

*Latrodectus* venom contains a rich cocktail of toxins (latrotoxins) and other biologically active substances, which affect the nervous system of victims. As such, the venom has served for decades as a source of important biological tools that have been used to dissect and study the molecular mechanisms of exocytosis in neurones and endocrine cells. Particularly, this extensive scientific exploration has created a wealth of knowledge about the structure, specificity and modes of action of *α*-latrotoxin (*α*-LTX), the principal venom component toxic to vertebrates (for reviews, see Rosenthal and Meldolesi, 1989; Ushkaryov, 2002).

The insecticidal components of *Latrodectus* venom (latroinsectotoxins, LITs), in turn, have been looked at keenly by the chemical industry as potential pesticides, but remain relatively poorly understood, partly because they are more numerous, labile and difficult to purify than their vertebrate-specific counterpart. However, LITs are very similar to *α*-LTX, both structurally and functionally, and their comparison to this well-studied toxin could give important clues as to the architecture and modes of action of the insect-specific toxins. The purpose of this review is to summarise the current knowledge regarding the structures and effects of LITs, complemented by the modern understanding of *α*-LTX, and to explore their potential as insecticides.

## 2 The complex venom

*Latrodectus* venom is produced by glandular cells in the spider's chelicerae. In a process called holocrine secretion, these cells disintegrate and their content is released into the lumen of the gland (Smith and Russell, 1966). This yields a complex mixture of toxins, enzymes and other cellular constituents, and the purification of specific active components from this venom can be challenging. High (and similar) molecular masses of LTXs and their propensity to oligomerise and aggregate further exacerbate the difficulty. Therefore, it is advantageous to limit the starting material for purification and physiological studies to the soluble contents of the venom gland lumen (see e.g. Majori et al., 1972). However, most initial studies used crude buffer extracts from venom glands, and only relatively recently did venom suppliers start producing venom by milking spiders. For example, Fauna Laboratories Ltd. has been milking captive black widow females by electrical stimulation of the venomous apparatus for a number of years now, leading to consistent isolation of a highly pure and potent *α*-LTX (Ashton et al., 2000). Another venom supplier, Alomone Labs Ltd., has joined suit and found that *α*-LTX isolated from milked venom was 10-fold more potent than the toxin extracted from venom glands (Meir, 2003).

Venom application to various tissue preparations led to the general observation (or implication) of a strong increase in neurotransmitter secretion. Over three decades ago, it was found that the venom was active in both insects and vertebrates (D’Ajello et al., 1969, 1971; Majori et al., 1972; Griffiths and Smyth, 1973), and that its action was not limited to acetylcholine (Cull-Candy et al., 1973), as had been originally hypothesised (e.g. Bettini, 1971; Majori et al., 1972).

Heating the venom resulted in loss of toxicity, indicating that its active components were proteins (e.g. D’Amour et al., 1936). This was confirmed by pre-treatment of crude gland extracts with antiserum, which completely blocked the toxicity in cockroaches, ruling out a major role for small molecular weight substances, such as serotonin (Majori et al., 1972). First attempts at purifying these proteins suggested the presence of several high molecular mass proteins, which seemed to act in a phylum-specific manner. The first purification by column electrophoresis on cellulose powder (Frontali and Grasso, 1964) gave three major fractions, of which two were toxic to house flies but had distinct effects, and one was active in guinea-pigs. Later, a fourth fraction was found to be toxic to crustaceans (Bettini, 1971). Further improvements in chromatographic matrices led to the purification by gel-filtration and subsequent ion-exchange chromatography (Frontali et al., 1976) of a 130-kDa protein specific to vertebrates, which was separated from the less pure fractions active in insects and crayfish. In another approach, semi-preparative electrophoresis of venom gland extract in native polyacrylamide gels yielded a ∼125-kDa protein band, active in cockroach nymphs and neuromuscular preparations but clearly containing other protein components (Ornberg et al., 1976). Interestingly, a set of 5-kDa proteins was also isolated by this method and shown to have a very rapid, but transient, paralytic activity in insects (Ornberg et al., 1976); since then, there have been no further reports regarding these potentially interesting peptides.

The vertebrate-specific toxin from *Latrodectus* venom was later termed *α*-LTX (Tzeng and Siekevitz, 1978), whereas a part-purified high molecular-mass insecticidal component was named *β*-LTX (Knipper et al., 1986). Subsequent systematic efforts of the Grishin group (Krasnoperov et al., 1990, 1991) have led to the purification and modern nomenclature (Grishin, 1998) of all currently known LTXs. To date, black widow spider venom has been found to contain seven proteins with neurotoxic activity. There are five insectotoxins: *α*, *β*, *γ*, *δ*, and *ε*-LIT, with respective molecular masses of 120, 140, 120, 110 and 110 kDa, one latrocrustatoxin, *α*-LCT (120 kDa), and one vertebrate toxin, *α*-LTX (130 kDa). *ε*-LIT is also highly toxic to *Caenorhabditis elegans* (Mee et al., 2004). Four LTXs have been cloned: *α*-LTX (Kiyatkin et al., 1990), *α*-LCT (Danilevich et al., 1999), *α*-LIT (Kiyatkin et al., 1993) and *δ*-LIT (Dulubova et al., 1996).

In addition, two low molecular weight proteins (known as LMWPs) usually co-purifying with *α*-LTX (Kiyatkin et al., 1992) have been identified and cloned (Gasparini et al., 1994; Volkova et al., 1995). LMWPs are structurally related to crustacean hyperglycemic hormones and have molecular masses of 8 and 9.5 kDa. Independently, another group also cloned LMWP and termed it latrodectin (Pescatori et al., 1995). These peptides are non-toxic by themselves (Gasparini et al., 1994; Kiyatkin et al., 1995; Volkova et al., 1995), but seem to increase the toxicity of the large LTXs (Grishin, 1998; Kiyatkin et al., 1995), probably by augmenting their affinity for the plasma membrane (Grishin et al., 1993).

Purified LTXs have been consistently reported to act in a strictly phylum-specific manner (e.g. Frontali et al., 1976; Magazanik et al., 1992; Grishin, 1998). For instance, pure *α*-LTX, even at a concentration of 2 μM, i.e. three orders of magnitude higher than its normal working concentration in mammals, was not toxic to *C. elegans*, while *ε*-LIT was highly potent in this nematode (Mee et al., 2004). Some reports have suggested that *α*-LTX might also act directly on insect synapses (Knipper et al., 1986; Umbach et al., 1998). However, the chromatographic fractions used in these works could contain some insectotoxins, in particular *δ*-LIT, which is difficult to remove completely from *α*-LTX preparations (Ashton et al., 2000). Only native polyacrylamide gel electrophoresis, which disrupts *δ*-LIT dimers but preserves the dimeric *α*-LTX, has enabled the purification of *α*-LTX free from *δ*-LIT (Ashton et al., 2000). When this pure toxin was tested in the *Drosophila* neuromuscular junction (NMJ), it was found to be ∼20-fold less potent than *α*-LTX purified by the conventional methods (J. Umbach, C. Gundersen, personal communication), while retaining full toxicity for mammals. Another possible explanation for *α*-LTX being active in the fly could be the presence of LMWPs, which are known to facilitate *α*-LTX action (Kiyatkin et al., 1995) and could lower its specificity. One of the best methods to assess the specificity of toxin/receptor interactions is the use of recombinant proteins, and this approach has again demonstrated the inactivity of *α*-LTX in insects and the potential ability of LMWPs to make this toxin active across phyla (Kiyatkin et al., 1995).

It must also be stressed that venom preparations from different species, or sub-species, of *Latrodectus* often give very different chromatographic profiles (cf. Frontali and Grasso, 1964; Frontali et al., 1976; Ashton et al., 2000; Graudins et al., 2001). There is also anecdotal evidence of local and even seasonal variations in venom contents (Keegan et al., 1960; Y. Ushkaryov, unpublished observations). However, the principal components of different venoms seem to be very similar or indistinguishable by SDS-gel electrophoresis and immunological analysis. For example, insectotoxins from even a distinct theridiid spider, *Steatoda paykulliana*, were shown to have the same immunological properties as the LITs from the *L. mactans* venom (Cavalieri et al., 1987). Also, an *α*-LTX-like protein has been detected in the venom of *Steatoda grossa* by western blotting using a polyclonal *α*-LTX antibody and shown to have similar, albeit weaker, activity in the vertebrate NMJ (Graudins et al., 2002).

## 3 Insectotoxins and their effects

### 3.1 Phenotypic manifestations

Behavioural aspects of insect latrodectism are obviously scarcely monitored, let alone reported, but venom effects on cockroach behaviour have been investigated (Franklin, 1988) and many symptoms were found to be similar to those described for humans. For instance, limb hyperextension and jerking in cockroaches correspond to muscle rigidity, motor restlessness, cramps, and increase in tendon flexes in humans. Quivering in cockroaches resembles fibrillation of groups of muscle fibres near the site of a bite in humans, often followed by trembling and clonic contractions. Cardiac block after a transient increase in the rate of heartbeat in *Periplaneta americana* is paralleled by tachycardia and hypertension in humans.

### 3.2 Toxicity

It is difficult to compare the published data on the toxicity of different LITs because preparations of variable purity have been tested on a diversity of insect species. The LD_50_ (micrograms of protein that cause death in 50% of animals, expressed per kg of body weight) for house flies (*Musca domestica*) of the first two semi-purified insecticidal fractions from black widow spider venom was 12 μg/kg (Frontali and Grasso, 1964). However, when five insectotoxins were later purified to homogeneity and tested on wax moth (*Galleria mellonella*) larvae (Krasnoperov et al., 1990), a similar value (15 μg/kg) was reported only for one protein (*α*-LIT). The other insectotoxins systematically studied in that work (Krasnoperov et al., 1990; Grishin, 1998) had lower toxicities: 25 μg/kg (*β*-LIT), 60 μg/kg (*δ*-LIT), 250 μg/kg (*γ*-LIT) and 1000 μg/kg (*ε*-LIT). Recombinant *δ*-LIT (Dulubova et al., 1996) was more toxic to house fly larvae (LD_50_ 10–50 μg/kg) than the toxin isolated from venom. One explanation for these differences in toxicity could be the distinct target specificities of LITs. Indeed, *ε*-LIT, while being almost inactive in wax moth larvae, is highly toxic to nematodes (LD_50_∼1–2 μg/kg). On the other hand, the LITs may be differentially sensitive to specific procedures used for their isolation. It is notable in this respect that the reported LD_50_ of even the best-studied and most stable LTX, *α*-LTX, ranges widely from 4.3 to 20 to 95 μg/kg (as determined, respectively, by Frontali and Grasso, 1964; Grishin, 1998; Frontali et al., 1976), probably reflecting protein inactivation during purification.

### 3.3 Different neurotransmitter systems

Physiological effects observed in insect tissue preparations on application of the whole venom or purified insecticidal fractions are consistent with a dramatic enhancement of transmitter secretion. Similar to the actions of *α*-LTX in vertebrates, LITs initially lead to a great increase in the frequency of glutamatergic and GABA-ergic miniature end plate potentials at insect NMJs and subsequent decline and cessation of asynchronous release (Griffiths and Smyth, 1973; Cull-Candy et al., 1973; Ornberg et al., 1976; Magazanik et al., 1992; Dulubova et al., 1996). NMJs from cockroach (Griffiths and Smyth, 1973; Ornberg et al., 1976), *Drosophila* (Broadie et al., 1995), locust (Cull-Candy et al., 1973; Dulubova et al., 1996) and blowfly (Magazanik et al., 1992) were studied and appeared to react in basically the same manner; effects of purified *α*-LIT (Magazanik et al., 1992) and *δ*-LIT (Dulubova et al., 1996) were similar to those of the whole venom. This vesicular release was dependent on the presence of intact SNARE proteins—synaptobrevin and syntaxin (Broadie et al., 1995). Ultrastructural studies demonstrated loss of synaptic vesicles from some (but not all) synapses, as well as vesicle clumping (Cull-Candy et al., 1973). In lobster NMJs (Fritz et al., 1980), such vesicle clumps were associated with the appearance of giant spontaneous potentials, or “giant minis”, thought to be due to release of clumped or internally fused vesicles. In addition, by acting through cardiac nerve ganglion and/or myocardial NMJs, the venom induced irregular heart beat and eventual “heart block” (Majori et al., 1972).

*Latrodectus* venom also affects the cholinergic sensory nervous system of insects. Venom application to the sixth abdominal ganglion from cockroach (*P. americana*), caused a massive release of acetylcholine from cercal sensory neurones, leading to depolarisation of somatodendritic membrane of giant interneurones in the ganglion (D’Ajello et al., 1969, 1971). Similar effects (Neri et al., 1965) were caused by partially purified insectotoxic fractions isolated by column electrophoresis (Frontali and Grasso, 1964).

At least in the case of cockroach ganglion preparations, the venom can apparently access and stimulate the insect central nervous system (Franklin, 1988). The sensitivity of insect central neurones to various fractions of black widow spider venom was further confirmed by monitoring release of acetylcholine from locust synaptosomes (Knipper et al., 1986).

### 3.4 Formation of membrane pores

One of the major molecular events in the action of *α*-LTX is the formation of membrane pores permeable to cations (Finkelstein et al., 1976), but this effect is negligible in tissues that lack *α*-LTX receptors. Therefore, in vertebrate neuromuscular preparations even whole black widow venom acts selectively on motor nerve terminals. In insect NMJs, in addition to the major presynaptic effect, the whole venom causes a clear, albeit minor, postsynaptic action (Griffiths and Smyth, 1973). In particular, the venom causes fluctuations of the muscle membrane potential almost immediately after its application, before there is any massive release of transmitter. A similar depolarisation of the muscle membrane was detected when pure *δ*-LIT was applied to *Drosophila* larval NMJs (J. Umbach, C. Gundersen, personal communication). These effects could be due to some venom components forming ion-permeable pores indiscriminately. Insectotoxins from the venom of a closely related spider *S. paykulliana* were indeed shown to create ion-permeable channels in artificial membranes (Cavalieri et al., 1987). Similar observations were later made on artificial bilayers using purified *α*-LIT (Shatursky et al., 1995; Magazanik et al., 1992) and recombinant *δ*-LIT (Dulubova et al., 1996). These channels/pores were highly conductive to Ca^2+^, and *α*-LIT appeared to have two binding sites for this cation (Shatursky et al., 1995).

Once the venom or individual LITs started to act, they could not be washed out (D’Ajello et al., 1969; Neri et al., 1965; Magazanik et al., 1992), corroborating the idea of toxin incorporation into the lipid phase. However, all the effects can be blocked by specific antisera (Majori et al., 1972; D’Ajello et al., 1969; Volkova et al., 1995), even when applied after the onset of the effect (Neri et al., 1965). This indicates that the toxins are not completely internalised and are still accessible from the extracellular space.

The hallmark of the vertebrate-specific *α*-LTX is its ability to induce transmitter exocytosis even in the absence of Ca^2+^, although in this case Mg^2+^ is absolutely required (Longenecker et al., 1970; Misler and Hurlbut, 1979; Meldolesi et al., 1983). This is also true for *α*-LIT and *δ*-LIT, as shown in blowfly (Magazanik et al., 1992) and locust (Dulubova et al., 1996) NMJs. In the latter work, a very small amount of transient secretion was also seen in the total absence of divalent cations, but only when the *δ*-LIT concentration was increased 1000-fold (Dulubova et al., 1996).

A detailed structural analysis of LITs is required in order to interpret physiological effects and understand the molecular mechanisms of these toxins.

## 4 Structure

### 4.1 Primary structure and sequence homology

**Fig. 1:**
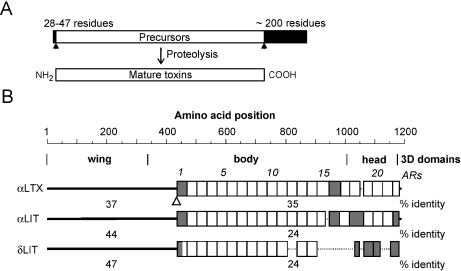
Comparison of latroinsectotoxins (LITs) with *α*-latrotoxin *α*-LTX. (A) All latrotoxins are post-translationally processed to yield mature, active toxins. Black arrowheads: potential sites for proteolysis of *α*-LTX, *α*-LIT and *δ*-LIT, which share the consensus sequence *K*/*R*−*Φ*_1–3_−*K*/*R*_0−1_−*R*↓, where *K* and *R* are lysines and arginines, respectively; *Φ* is a hydrophobic/aromatic amino acid and ↓ denotes the cleaved peptide bond. (B) Domain organisation of *α*-LIT and *δ*-LIT in comparison to *α*-LTX. Top, the amino acid position scale and 3D domain organisation of *α*-LTX (*wing*/*body*/*head*). The amino acid sequences, presented diagrammatically below, were aligned using a method best suited for sequences related by descent (Hein, 1990). The unique N-terminal domains are depicted as solid black lines. ARs are shown as boxes and are numbered above the *α*-LTX structure; sequences not recognised by the PFAM database algorithm are coloured grey. Gaps in the alignment are indicated with dots. The four-residue insertion in the *α*-LTX^N4C^ mutant (see text) is denoted by an open arrowhead. Numbers between the diagrams indicate percentage identities between the respective domains (numbers below the *δ*-LIT structure are relative to *α*-LTX).

Similarly to *α*-LTX, LITs are synthesised as large precursor molecules (Fig. 1A) (Kiyatkin et al., 1990, 1993; Dulubova et al., 1996) which are not toxic (Kiyatkin et al., 1995; Dulubova et al., 1996). Upon disintegration of secretory cells, the protoxins undergo proteolytic processing in the glandular lumen. This involves a furin-like protease, which cleaves similar sequences at both the N- and C-termini of all LTXs (Fig. 1A). The cleavage results in the removal of a 28- to 47-residue N-terminal leader sequence and of about 200 residues from the C-terminus. As a result of this processing, LTXs become activated (Kiyatkin et al., 1995; Dulubova et al., 1996).

The mature versions of all LTXs consist of two major parts: the N-terminal domain, which has no substantial homology to any known proteins (termed herein “unique”), and the C-terminal part, which harbours multiple ankyrin repeats (ARs, Fig. 1B). ARs are found in hundreds of proteins with vastly different functions—from scaffolding to transcription control to ion conductance—and are thought to mediate stabilising protein–protein interactions (Sedgwick and Smerdon, 1999). In LTXs, the number of ARs varies from 13 in *δ*-LIT to 20 in *α*-LTX (22, including two imperfect repeats; see Fig. 1B), and these repeats appear to organise the C-terminal region into a compact globular structure (see below).

In addition to having the same principal architecture, the LTXs share strong sequence similarities. Thus, overall sequence identity of *α*-LTX, *α*-LIT and *δ*-LIT ranges from 32% to 35%, with slightly higher homology in the unique N-terminal region (37–47%) (Fig. 1B). As a rule, each individual AR shows much less similarity to its neighbour repeats (on average, 24%) than it does to the respective ARs in other LTXs, where sequence identity is often 50%. This suggests that the repeating AR structure of LTXs arose at an early stage of evolution, probably before these intracellular proteins became neurotoxins with strict target specificities. It also indicates that the multi-AR structure of LTXs is subject to high selective pressure. In this context, it seems plausible that the presence of fewer ARs in *δ*-LIT than in the *α*-LTX and *α*-LIT is the result of a deletion that occurred during subsequent evolution.

### 4.2 Three-dimensional structure

**Fig. 2:**
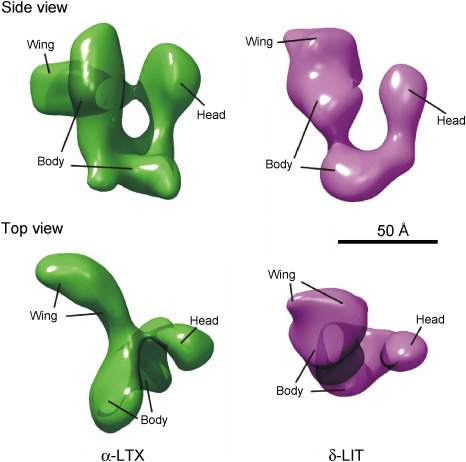
The 3D structures of *α*-LTX and *δ*-LIT monomers. Comparison of the side and top views of the *α*-LTX monomer obtained by single-particle analysis of cryo-electron micrographs (Orlova et al., 2000) with similar views of the *δ*-LIT monomer, reconstructed by single-particle analysis of negative-stain electron micrographs. The domains of *α*-LTX are marked as in (Orlova et al., 2000); tentative domain assignments in the *δ*-LIT reconstruction are based on the interactive overlap of the 3D maps, fitting of ARs and volumetric analysis. Both volumes were low-pass filtered to 20 Å. (J. Nield, R. Abbondati, B. Odier, A. Rohou, Y. Ushkaryov, unpublished results).

So far, attempts to crystallise LTXs have not been successful, but the structure of the vertebrate-specific *α*-LTX has been studied by electron cryo-microscopy (Orlova et al., 2000). This method attains a lower resolution compared to X-ray crystallography but it does not depend on the ability of a protein to form crystals and, crucially, permits the visualisation of proteins instantaneously frozen in their native state in vitrified ice. The three-dimensional (3D) structure reveals the toxin to be a stable dimer that further assembles into tetramers. The structure of the *α*-LTX monomer alone, as extracted from the 3D reconstruction of the tetramer (Fig. 2), demonstrates that the molecule contains three rather than two (as inferred from the amino acid sequence, Fig. 1B) distinct domains: the *wing*, the *body* and the *head*. Probing with N-terminal antibodies and fitting crystal structures of ARs into the 3D model of the toxin indicated that the *wing* constitutes a large part of the unique N-terminal sequence, while the *body* and the *head* together contain all the ARs. The *body* combines about one quarter of the unique N-terminal domain and the first 15–16 ARs, while the *head* includes ∼4.5 C-terminal ARs (see Fig. 1B). Despite the seemingly narrow link between the *wing* and the *body*, their relative orientation always remains constant, probably owing to a multi-point connection in the link area. The *head* is bent back towards the *body* and may be attached to the *wing* via a disulphide bridge (Orlova et al., 2000).

Very little is known about the 3D structures of LITs. However, their amino acid sequences are 33–35% identical to that of *α*-LTX and largely consist of repeated elements (ARs) that always produce the same 3D fold (Mosavi et al., 2004). It would seem likely, therefore, that all LTXs have very similar folds. Recently, structural studies of *δ*-LIT were initiated in order to allow for comparison between toxin structures and, thus, aid in the understanding of their phylum specificity and molecular mechanism of action. Preliminary results (J. Nield, R. Abbondati, B. Odier, Y. Ushkaryov, unpublished), including a ∼20 Å-resolution reconstruction of the *δ*-LIT monomer (Fig. 2), suggest that *δ*-LIT indeed has a very similar architecture to that of *α*-LTX. Most strikingly, despite low-sequence homology between their C-termini, *δ*-LIT contains a domain similar in shape to the *head* of *α*-LTX, although slightly smaller. Volumetric measurements suggest that this might correspond to the three imperfect C-terminal repeats that are seen to align against the *α*-LTX *head* (see Fig. 1). *δ*-LIT contains fewer ARs in the C-terminal part of its *body* than *α*-LTX does. Accordingly, the equivalent domain in the *δ*-LIT reconstruction appears to be somewhat smaller, constricted in the middle and not immediately recognisable. However, molecular fitting shows that it could still house ARs 1–13. The N-terminal domain, an apparent correlate of the *α*-LTX *wing*, is not clearly separated from the ARs of the *body* in this preliminary reconstruction and, instead of protruding sideways, as in *α*-LTX, it might simply extend the *body* domain.

### 4.3 Oligomerisation

**Fig. 3:**
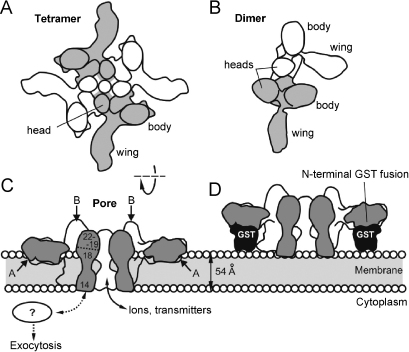
Dimers and tetramers of *α*-LTX and the mechanism of membrane pore formation. (A, B) Top views of the two major multimeric species of *α*-LTX. The monomers are coloured alternately. Both dimers and tetramers are able to bind receptors but only the tetramer forms integral membrane pores. (C) Cut-open side view of the *α*-LTX tetramer inserted into a lipid bilayer. The pore in the centre of the tetramer is permeable to ions and cytosolic neurotransmitters. Approximate positions of trypsin cleavage sites A and B (see text) are indicated with arrows. The locations of ARs 14–22 are shown as numbers in the cross-section of one monomer. (D) A predicted steric hindrance mechanism which may prevent membrane pore formation by N-terminal fusion constructs of *α*-LTX. Sections through glutathione-S-transferase (GST) are shown to scale.

One of the most important features of *α*-LTX is arguably its ability to form dimers and tetramers (Fig. 3A, B). Analysis of the mechanisms and implications of this oligomerisation can throw light onto the structure and mode of action of all insectotoxins, including LITs.

*α*-LTX dimers are asymmetric, with the *head* of one monomer binding in between the *wing* and the *body* of the other and the *wings* extending outwards at a 90° angle in one plane (Fig. 3B) (Orlova et al., 2000). The dimers can spontaneously associate into cyclical C4-symmetric tetramers (Fig. 3A). *α*-LTX monomers appear to have a very high affinity for each other and re-associate even after boiling in SDS (Ashton et al., 2000). Its oligomers exist in a dynamic equilibrium that shifts towards dimer formation after treatment with EDTA or La^3+^ and towards tetramers on addition of Ca^2+^ or Mg^2+^ (Ashton et al., 2000). Tetramers are disrupted by non-denaturing detergents and native electrophoresis (Ashton et al., 2000) but are greatly strengthened by low amounts of amphipathic reagents (M. Rahman, C. Manser, Y. Ushkaryov, unpublished observation).

It is important to note that oligomerisation of *α*-LTX is functionally very significant: tetramers have been directly observed inserting into a lipid membrane and harbouring a central channel, lined by the C-terminal *heads* (Fig. 3A, C) (Orlova et al., 2000). Furthermore, when tetramerisation is inhibited (e.g. by EDTA, La^3+^ or mutagenesis), the “classical”, pore-mediated effects of the toxin are no longer observed (Ashton et al., 2000, 2001; Capogna et al., 2003) (see also below). In other words, only the tetramer of *α*-LTX seems to form pores and can conceivably lead to uncontrolled release from target cells.

LITs probably undergo similar rearrangements, and some features of LITs (e.g. appearance as multiple peaks in chromatographic profiles and propensity to precipitate *en masse* out of solution; Y. Ushkaryov, unpublished) can be explained by oligomer formation and aggregation. In fact, *δ*-LIT has been shown to exist as a mixture of monomers (as in Fig. 2) and dimers (Ashton et al., 2000). Even though *δ*-LIT dimers are much weaker than those of *α*-LTX and disintegrate on the application of an electric field, they, too, have been found to assemble into stable tetramers (Ashton et al., 2000). As for *α*-LTX, formation of *δ*-LIT tetramers is enhanced by the presence of amphiphatic molecules, to the extent that such tetramers remain stable under native electrophoretic conditions (Ashton et al., 2000). It is possible that membrane lipids have similar effects on the oligomeric state of *δ*-LIT and other LITs. However, in contrast to *α*-LTX, *δ*-LIT does not require Ca^2+^ or Mg^2+^ for tetramerisation.

LITs are strikingly similar to *α*-LTX both structurally and functionally, and, given that they also form membrane pores, it seems highly likely that LITs must also oligomerise in order to form pores. However, oligomeric structures of LITs are poorly understood and require further study.

## 5 Modes of action

### 5.1 Cation-permeable pores

Formation of cation-permeable membrane pores in biological and artificial membranes has been demonstrated for *α*-LIT and *δ*-LIT (Shatursky et al., 1995; Dulubova et al., 1996), as well as for *α*-LTX (Finkelstein et al., 1976). Owing to their extensive structure conservation, the mechanism of this action is probably very similar in *α*-LTX and *α*-LIT. However, only in *α*-LTX has pore formation been studied in molecular terms. Therefore, we will summarise here the existing knowledge about *α*-LTX pores, with reference, where possible, to LITs.

Channel formation is one of the main (and certainly the most toxic) effects of the vertebrate-specific *α*-LTX. It leads to influx of extracellular cations (Ca^2+^ and Na^+^). Because the channel is also permeable to water (Krasilnikov and Sabirov, 1992), it causes swelling of nerve terminals (e.g. Pennuto et al., 2002). Additionally, the pores mediate efflux of cytosolic cations, including K^+^, and even small molecular weight substances such as neurotransmitters and ATP (McMahon et al., 1990; Adam-Vizi et al., 1993; Deri et al., 1993; Hurlbut et al., 1994; Davletov et al., 1998; Ashton et al., 2000). Any one of these effects would be detrimental to the membrane potential, energy balance and/or structural integrity of neuronal cells, but their combination causes terminal degradation and death (e.g. Robbins et al., 1990).

### 5.2 Toxin domains involved in pore formation

It is important to establish which domain/s of a typical LTX is/are involved in membrane penetration. This knowledge could allow the identification of a minimal LIT fragment that would still cause the desired insectotoxic effect, whilst being commercially viable and deliverable to insects.

The effects of *α*-LTX pore formation in biological systems are only observed in the presence of Ca^2+^ or Mg^2+^. It is not surprising, therefore, that the *α*-LTX tetramer (which is induced in those conditions) is the only *α*-LTX species that has been shown to insert into membranes (Orlova et al., 2000). These direct observations have shown, beyond doubt, that the *body* domains assemble into the “bowl-like” base of the *α*-LTX tetramer, and that it is this base that penetrates the lipid bilayer (Fig. 3C). In this configuration, the N-terminal *wings* do not penetrate into the lipid phase vertically but lie splayed atop the bilayer, being slightly embedded into the layer of polar lipid heads. The rigidity of the *wing*-*body* connection also makes it unlikely that the *wings* subsequently fold and pierce the membrane to interact directly with cytosolic components of the exocytotic machinery, as was previously hypothesised (Khvotchev and Sudhof, 2000).

Some clues regarding the functions of the *wing* and other domains of *α*-LTX have emerged from studies of *α*-LTX mutants expressed in insect cells (Ichtchenko et al., 1998; Volynski et al., 2003) and bacteria (Li et al., 2005). For example, cysteine to serine substitutions in the *wing* (as well as reducing conditions) abolish receptor binding and secretion stimulation (Ichtchenko et al., 1998), concurrent with the hypothesis that this domain may be involved in receptor binding (Orlova et al., 2000).

Another *α*-LTX mutant, LTX^N4C^ (Ichtchenko et al., 1998), has a four-residue insert immediately before the first AR (open arrowhead in Fig. 1B). It is noteworthy that this insert is unlikely to affect the structure of the N-terminal *wing* directly, since it occurs much later in the sequence, in the *body* domain. In this mutant, the relative orientation of *bodies* and *wings* has been found to be altered (Y. Ushkaryov, unpublished observation), and tetramerisation abolished (Volynski et al., 2003). In turn, its inability to tetramerise means that LTX^N4C^ cannot permeate membranes and form pores (Khvotchev and Sudhof, 2000; Volynski et al., 2003; Capogna et al., 2003). Importantly, rather than implicating the N-terminal in the membrane insertion, the work on LTX^N4C^ actually shows that membrane insertion correlates with tetramerisation (Ashton et al., 2001).

In more recent mutagenesis experiments (Li et al., 2005), N-terminal GST-tagging of the *α*-LTX *wing* hampered pore formation, while not interfering with receptor binding, and this fact was taken to confirm the insertion of the N-terminus into the membrane. However, this result actually corroborates the tetrameric model of pore formation. As demonstrated using a specific antibody (Orlova et al., 2000), the tip of the *wing* is formed by a loop of internal sequence, and the NH_2_-terminus localises away from the tip. The presence of a bulky GST moiety (∼27 kDa), attached near the middle of the 36-kDa rigid *wing*, likely obstructs the apposition of the *wing* against a lipid bilayer and in this way prevents the full insertion of the tetramer into the membrane (Fig. 3D).

The same authors (Li et al., 2005) also carried out several truncations of the C-terminal ARs, which make up the *head* of each monomer (Fig. 3C). As the *heads* both hold the tetramer together and line the central channel, any perturbations of their structure would be predicted to change the properties of the channel or even lead to tetramer disruption. This prediction was completely borne out by the results (Li et al., 2005): when only the most C-terminal half-repeat was removed, the pore became much more conductive than in wild type *α*-LTX, whereas all further AR deletions led to partial or complete loss of the pore-forming ability. Significantly, the *wing* in these pore-incapable mutants was left intact. This unequivocally rules out any direct N-terminal insertion as the means of pore formation and confirms the model (Fig. 3C and Orlova et al., 2000) proclaiming pores to be made by tetramers of *α*-LTX.

This model is also consistent with protease protection assays used to identify parts of the protein that are shielded from the extracellular solvent by the membrane and, thus, are probably involved in pore formation. Upon *α*-LTX binding to receptor-expressing membranes, a significant proportion of toxin molecules became partially or fully protected against proteases (Khvotchev and Sudhof, 2000). This effect appeared to require protein transition into the lipid phase (Krasil’nikov et al., 1985). There are two major trypsin cleavage sites in membrane-associated *α*-LTX (Khvotchev and Sudhof, 2000) (Fig. 3C). Site A is located in the unique N-terminal domain and is exposed when *α*-LTX is not fully integrated into the membrane, whilst site B (found near the first AR) appears to be exposed at all times (Khvotchev and Sudhof, 2000). These data correlate elegantly with our current structural understanding of the *α*-LTX fold (Fig. 3C), which places site A in the *wing*, and site B near the top of the *body*, the region furthest from the membrane in the inserted tetramer.

An insight into the basic molecular topology of *α*-LTX by electron cryo-microscopy indicates that the N-terminal domains (*wings*) are oriented in precisely the same manner in every tetramer and are rigidly attached to the *body* at ∼90° to each other (Fig. 3A, B) (Orlova et al., 2000; A. Rohou, unpublished observations). Therefore, if the N-terminal region of *α*-LTX were to penetrate directly through the membrane, then only one *wing* in any given dimer or tetramer would be able to carry out this hypothetical role. The rest of the *wings*, in the manner of a Chinese throwing star sunk in a target, would not contact the membrane, being functionally redundant. Overall, the balance of evidence does not support the idea that pore formation is caused by insertion of the *α*-LTX N-terminal domain into the membrane.

In the case of *δ*-LIT, and in light of our preliminary structural characterisation, it would seem even more unlikely that the N-terminal domain is involved in membrane penetration, as it appears to be completely attached to the top of the *body* (Fig. 2). We speculate that, similar to *α*-LTX, *δ*-LIT forms ion-permeable pores by assembling into tetramers, since this rearrangement is facilitated by amphipathic molecules and possibly lipids.

### 5.3 What do LTXs need to make pores?

Both *α*-LTX and LITs have been shown to produce membrane pores in the membrane of receptor-expressing cells (e.g. Volynski et al., 2000; Magazanik et al., 1992; Dulubova et al., 1996), but also in artificial lipid bilayers (Finkelstein et al., 1976; Dulubova et al., 1996). However, this effect seems somewhat paradoxical: if the toxins can form pores in lipid bilayers, then it is impossible to justify their tissue and phylum specificity. The explanation likely lies in the efficiency of membrane insertion. The latter depends on the type of membrane, on the presence of receptors that facilitate the toxin attachment to the membrane, and on the toxin interaction with auxiliary proteins from the venom (like LWMP), which seem to promote the interaction with membranes (Grishin et al., 1993).

Interestingly, *δ*-LIT pore formation appears less dependent on the presence of specific receptors. For instance, *δ*-LIT can form pores not only in artificial lipid bilayers, but also in the membrane of muscle cells (Dulubova et al., 1996; J. Umbach, C. Gundersen, pers. commun.), suggesting a lack of neuronal selectivity. On the other hand, very little is known about the specific biological activity of *δ*-LIT.

### 5.4 Receptors

**Fig. 4:**
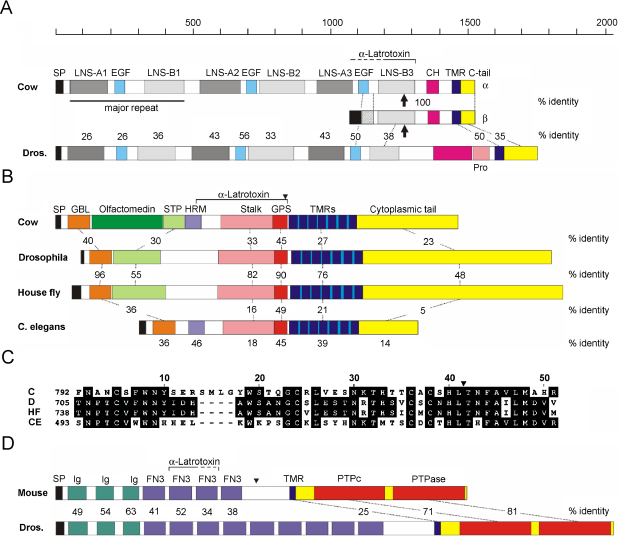
Evolutionary conservation of the three types of *α*-LTX receptors. (A) Neurexins in cow and *Drosophila*. Vertebrates possess three neurexin genes, each of which encodes a long (*α*) and a short (*β*) form of neurexin. (B) Three latrophilins are found in vertebrates (LPH1 from the cow, *Bos taurus*, is shown), whereas only one form is present in insects (*Drosophila* and house fly, *M. domestica*). The nematode (*C. elegans*) LPH is illustrated for comparison because it binds *ε*-LIT from *Latrodectus* venom (Mee et al., 2004). (C) Multiple sequence alignment of the GPS domains from cow, C; *Drosophila*, D; housefly, HF; and *C. elegans*, CE. Identical residues are highlighted. (D) Receptor-like protein tyrosine phosphatase *σ* (PTP*σ*) in mouse and *Drosophila*. (A–D) Percentage identities are indicated by numbers between the respective domains (values below the *C. elegans* structure correspond to the cow sequence). The minimal protein regions required for *α*-LTX binding are indicated by square brackets above the diagrams. Domain names are abbreviated as follows: CH, O-linked carbohydrate domain; EGF, epidermal growth factor-like domains; FN3, fibronectin III-like domain; GBL, galactose-binding lectin-like domain; GPS, G-protein-coupled receptor proteolysis site; HRM, hormone receptor motif; Ig, immunoglobulin-like domain; LNS, laminin G-domain/neurexin/sex hormone binding protein repeat; SP, signal peptide; STP, serine/threonine/proline-rich region; Pro, proline-rich region; PTPase, protein tyrosine phosphatase; TMR, transmembrane region. Black arrows in A denote the site of alternative splicing #4 within the last LNS repeat; small black arrowheads in B and D indicate the sites of constitutive proteolysis.

In order to form pores efficiently, most LTXs (perhaps with the exception of *δ*-LIT) require membrane receptors (Hlubek et al., 2000; Van Renterghem et al., 2000; Volynski et al., 2000); the latter also determine the specificity of the toxins’ action. Receptors for LITs have yet to be isolated, but considering the high-sequence conservation between *α*-LTX and *α*-LIT, it seems plausible that their receptors could be similar. Under these circumstances, the complete sequences of some insect genomes provide an invaluable resource for the identification of insect orthologues of *α*-LTX receptors. However, quite uniquely among neurotoxins, *α*-LTX binds to not just one but three different classes of neuronal receptors, which (in order of their discovery) are neurexin I*α* (Petrenko et al., 1990; Ushkaryov et al., 1992), latrophilin 1 (Davletov et al., 1996; Krasnoperov et al., 1996) and receptor-like protein tyrosine phosphatase *σ* (PTP*σ*) (Krasnoperov, V.G. et al., 2002; Fig. 4). These three receptor classes are found also in insects and are described below.

#### 5.4.1 Neurexins

In vertebrates, neurexins are a family of highly variable neuronal cell surface receptors (Fig. 4A) (Petrenko et al., 1990; Ushkaryov et al., 1992). They are transcribed from three homologous genes, each controlled by two independent promoters, thus giving rise to six major forms: three long (*α*) and three short (*β*) neurexins (for a review see Missler et al., 1998). The diversity of vertebrate neurexins is further enriched by extensive alternative splicing at five sites, where independent combinations of inserts may produce hundreds or even thousands of isoforms (Missler and Sudhof, 1998). All neurexins have a single transmembrane (TMR) domain and a very short cytoplasmic tail. In *α*-neurexin, the large extracellular sequences are composed of six LNS domains (for laminin G/neurexin/sex hormone-binding globulin). Three pairs of LNS domains, with epidermal growth factor (EGF)-like repeats in the middle of each pair, form three “major repeats” (Fig. 4A). In contrast, *β*-neurexins possess only one LNS domain. In all neurexins a region rich in threonines and serines separates the LNS domains from the TMR and provides multiple sites for extensive O-linked glycosylation.

Only neurexin I*α* binds *α*-LTX with high affinity, and this interaction strictly requires Ca^2+^. The binding site has not been specifically mapped, but several lines of evidence implicate the third major repeat. In particular, the toxin binding to neurexin I*α* occurred only in the absence of an insert at the splice site 4 located in the LNS-B3 domain (Davletov et al., 1995). A mutant neurexin I*α* lacking this domain (together with LNS-B2 and -A3) did not bind *α*-LTX, nor did neurexin I*β* containing LNS-B3 only (Davletov et al., 1995). Later, it was demonstrated that, under very mild conditions, LNS-B3 domains of all three *β*-neurexins (I*–*III*β*) may actually be able to interact with *α*-LTX (Sugita et al., 1999). Thus, it is plausible that the LNS-B3, whilst itself mediating only a weak interaction, determines whether the binding is at all possible and that other domain(s) upstream of LNS-B3—probably LNS-A3 (Li et al., 2005)—provide additional binding sites that account for the strong binding of *α*-LTX to neurexin I*α*.

The cytoplasmic tails of neurexins contain a C-terminal four-amino acid PDZ-interaction site that binds to scaffolding proteins CASK (Hata et al., 1996) and Mint (Biederer and Sudhof, 2000). These two proteins bind each other and Ca^2+^ channels; Mint also interacts with Munc18, a protein involved in the regulation of vesicle exocytosis (Verhage et al., 2000). Extracellular domains of *β*-neurexins bind neuroligins on the postsynaptic membrane, and this interaction is important for synapse formation (Scheiffele et al., 2000; Boucard et al., 2005). Even though *α*-neurexins have the same C-terminal sequences as *β*-neurexins, they are not involved in synaptogenesis but play an important role in organising Ca^2+^ channels in presynaptic nerve terminals. All these interactions are essential for synapse establishment, maintenance and long-term regulation, but they seem unlikely to underlie a massive and relatively fast response induced by *α*-LTX. Rather, the functionality of neurexins as *α*-LTX receptors (Sugita et al., 1999) is most easily explained in terms of membrane pore formation by the wild type toxin used in that work.

Interestingly, no alternative promoters or splicing events have been found in *Drosophila* neurexin (Tabuchi and Sudhof, 2002), indicating that insects express only one *α*-neurexin and no *β*-neurexins. Overall *Drosophila* neurexin is 33% identical to bovine neurexin I*α*, although the homology is much higher within the LNS A3 domain implicated in toxin binding. Thus, it is possible that *Drosophila* neurexin could provide binding sites for some LITs.

#### 5.4.2 Latrophilin

Latrophilin (Davletov et al., 1996; Lelianova et al., 1997), or CIRL (for Ca^2+^-independent receptor of LTX; Krasnoperov et al., 1996, 1997), is a large heptahelical receptor. It comprises three major domains (Fig. 4B): (1) a long glycosylated extracellular domain, (2) seven hydrophobic TMRs, and (3) a long cytoplasmic tail. The ectodomain contains regions of homology to: galactose-binding lectin (GBL); the surface-attached extracellular matrix protein olfactomedin (Snyder et al., 1991; Loria et al., 2004); a hormone receptor motif (HRM) found also in other G-protein-coupled receptors (GPCRs), where it is probably involved in ligand binding; a “Stalk” domain important for proteolytic cleavage (Chang et al., 2003; see also below); and the actual GPCR proteolysis site (GPS). The cytoplasmic region is phosphorylated and palmitoylated, and contains sequences mediating interactions with intracellular proteins. The seven TMRs are similar to the corresponding regions of the secretin/calcitonin receptor family, GPCRs that bind peptide hormones and induce release of various substances (Harmar, 2001).

Despite these similarities, latrophilin actually represents a novel family of GPCRs. The members of this group, termed “long N-terminus, group B” (LNB) (Hayflick, 2000; Stacey et al., 2000) or “adhesion” (Fredriksson et al., 2003) receptors, have an unusual architecture revealed only recently (Gray et al., 1996; Krasnoperov et al., 1997; Volynski et al., 2004). All LNB receptors contain a cell adhesion-like extracellular N-terminal domain and a signalling (seven-TMR) C-terminal domain (Hayflick, 2000; Stacey et al., 2000). Intriguingly, these receptors are post-translationally cleaved at the GPS (Krasnoperov et al., 1997) (Fig. 4C) into the N- and C-terminal fragments, which correspond to the two functional domains. This constitutive proteolytic cleavage, occurring in the endoplasmic reticulum, is a prerequisite of surface delivery (Krasnoperov, V. et al., 2002; Volynski et al., 2004). The GPS is localised upstream of the first TMR, yet the ectodomain is not released into the medium, apparently because it is attached to the membrane by an uncharacterised hydrophobic anchor (Volynski et al., 2004). Following delivery to the plasma membrane, the two receptor fragments are able to dissociate and behave as independent cell-surface proteins (Volynski et al., 2004). Re-association of the fragments is facilitated by *α*-LTX binding to the N-terminal fragment, and this correlates with signal transduction, which includes activation of G-protein and phospholipase C and subsequent release of Ca^2+^ from intracellular stores (Volynski et al., 2004).

There are three latrophilins in vertebrates (numbered 1–3) (Ichtchenko et al., 1998, 1999; Matsushita et al., 1999). Of these, only latrophilin 1 binds *α*-LTX with high affinity, while latrophilin 2 has a 10-fold weaker affinity. In both cases the binding is Ca^2+^-independent. The site of toxin binding has been mapped in latrophilin 1 and shown to encompass a large area comprising the HRM, Stalk and GPS domains (Krasnoperov, V.G. et al., 2002).

*Drosophila* genome contains several genes-encoding proteins that belong to the LNB family. Of these, only the CG8639 gene product is structurally similar to latrophilin. Although lacking the olfactomedin domain and HRM motif, it contains the stalk and GPS domains present in the *α*-LTX-binding site of vertebrate latrophilin (Fig. 4B, C). A very similar gene has been found in the house fly (Andreev et al., 2005) (Fig. 4B). Overall sequence identity of bovine and insect latrophilins is 22%, whereas the *Drosophila* and house fly latrophilins are 60% identical.

Nothing is currently known about the LIT-binding ability of insect latrophilins, and it may be interesting to consider another invertebrate latrophilin homologue from *C. elegans* (Mee et al., 2004; Willson et al., 2004). The nematode protein, encoded by the B0457.1 (or *lat-1*) gene, lacks the olfactomedin-like region but contains a complete set of domains required for toxin binding (HRM, Stalk and GPS). In that respect and in terms of sequence homology, this receptor bears a stronger resemblance to vertebrate latrophilin than to its insect orthologues (Fig. 4). However, it was found to mediate the lethal effect of *Latrodectus* venom by binding one of insectotoxins (presumably *ε*-LIT) (Mee et al., 2004). Although *C. elegans* also expresses neurexin and PTP*σ* homologues (see below), only the knockdown of the latrophilin gene rendered the worms resistant to *ε*-LIT (Mee et al., 2004). Interestingly, the worm latrophilin mediates the action of an anthelmintic drug, an octadepsipeptide called emodepside, which causes paralysis in nematodes (Willson et al., 2004). Analysis of various *C. elegans* mutants has proven that the action of emodepside is mediated by the coupling of latrophilin to G*α*q protein and then phospholipase C-*β* (PLC). The product of the PLC activity, inositol-trisphosphate, stimulates release of Ca^2+^ from intracellular Ca^2+^ stores, known to be important for *α*-LTX activity in mammals (Davletov et al., 1998; Capogna et al., 2003). In addition, the effect of emodepside involves unc13, a protein involved in control of vesicular exocytosis and activated by diacyl glycerol, another product of PLC. These data suggest that latrophilin homologues that possess the HRM domain can mediate signalling induced by LTXs, but it remains unclear whether insect latrophilin, which lacks this domain, can interact with any LIT.

#### 5.4.3 Receptor-like PTP*σ*

PTP*σ* is another neuronal cell surface receptor implicated in Ca^2+^-independent *α*-LTX binding (Krasnoperov, V.G. et al., 2002) (Fig. 4D). Its insect homologue is very similar to the vertebrate protein, with an overall sequence identity of 53%. The extracellular domain of this receptor contains three immunoglobulin-like (Ig) repeats and four fibronectin type III (FN3) repeats. Both these repeats are found in many classes of proteins and are thought to engage in protein–protein and receptor–ligand interactions, regulating cell adhesion and differentiation. Only the second and possibly third FN3 repeats are involved in binding *α*-LTX (Krasnoperov, V.G. et al., 2002) (Fig. 4D). A single TMR links the ectodomain to the cytosolic tail, which contains two tyrosine-specific protein phosphatase domains. It seems that the first has enzymatic activity, while the second is probably inactive and may be involved in binding phosphorylated tyrosine residues and/or regulating receptor activity (Pot et al., 1991).

Similar to latrophilin, PTP*σ* is proteolysed intracellularly upstream of the TMR to yield two subunits, the ectodomain and the membrane-bound C-terminal fragment (Aicher et al., 1997). The remaining extracellular portion of the C-terminal fragment, however, is much larger than the respective part in latrophilin (115 versus 19 residues). In PTP*σ*, it provides the only link tethering the ectodomain non-covalently to the plasma membrane, while in latrophilin there appears to be an additional modification anchoring the ectodomain in the membrane.

### 5.5 Receptor-mediated signalling

Because these receptors have principally different structures and physiological functions but seem to mediate the toxin's effect equally well (Sugita et al., 1999; Krasnoperov, V.G. et al., 2002), at least some major actions of *α*-LTX must be independent of the receptor structure. The most obvious and powerful mechanism of this type is the *α*-LTX pore formation described above: while receptors facilitate pore formation, they do not make a physical part of the pore, nor do they regulate its activity (Van Renterghem et al., 2000; Volynski et al., 2000; Hlubek et al., 2000).

Toxin–receptor interactions can potentially also stimulate intracellular signalling, especially when the toxins bind to latrophilin or PTP*σ*, both of which are clearly capable of signal transduction. This signal may conceivably lead to substantial increases in transmitter exocytosis. However, it is equally clear that any signalling, even if it leads to receptor over-stimulation, is based on physiological mechanisms and cannot have such a major, continuous and detrimental effect on secretory activity as that of a transmembrane pore. Moreover, the presence of toxin pores can make it impossible to measure or even detect the effects of signal transduction.

From that point of view, it is interesting to consider the LTX^N4C^ mutant already mentioned in this review. This mutant was initially thought to be inactive (Ichtchenko et al., 1998), but the methods used to monitor neurotransmitter release in that work were mostly sensitive to pore formation. Subsequent experiments (Ashton et al., 2001; Volynski et al., 2003; Capogna et al., 2003) revealed that this mutant completely lacks pore-forming activity but not affinity for the receptors. Consistent with the idea that the interaction of *α*-LTX with latrophilin and/or PTP*σ* can induce signal transduction, LTX^N4C^ is still able to trigger a strong increase in transmitter release measured both biochemically and electrophysiologically (Ashton et al., 2001; Volynski et al., 2003; Capogna et al., 2003). This effect is not grossly detrimental to neuronal cells and can last for many hours, in stark contrast to the action of the wild type, pore-forming *α*-LTX, which rapidly leads to the degradation of exocytotic activity. When probed pharmacologically, the signalling caused by LTX^N4C^ involved the activation of phospholipase C and release of Ca^2+^ from intracellular stores (Davletov et al., 1998; Capogna et al., 2003) and the same type of signal transduction was directly attributed to latrophilin in *C. elegans* (Willson et al., 2004) and in a model system (latrophilin-transfected neuroblastoma cells; Volynski et al., 2004).

Thus, *α*-LTX can clearly induce receptor-mediated intracellular signal transduction resulting in intense synaptic activity. If LITs trigger a similar activity by binding to insect receptors, they could lead to abnormal behaviours in targeted insects and potentially to their death, but the toxic effect would still be much milder than that of a pore-forming LIT. In addition, when considering LITs or their derivatives as potential ligands to over-stimulate the receptors, one must keep in mind that the simple binding may not be sufficient for signal initiation. Active ligands must meet a set of strict structural criteria, which, among other things, probably include receptor dimerisation, but can only be fully understood after further in-depth structural studies.

### 5.6 Partial toxin internalisation

One of the most enigmatic aspects of LTXs (*α*-LTX, *α*-LIT and *δ*-LIT) remains their ability to cause massive vesicular release in the absence of extracellular Ca^2+^, although Mg^2+^ is strictly required (Misler and Hurlbut, 1979; Ceccarelli and Hurlbut, 1980; Magazanik et al., 1992; Dulubova et al., 1996). To account for this effect, a search has been underway for many years for a mechanism, which stimulates exocytosis but does not require Ca^2+^ influx. Many candidate mechanisms have been put forward, none of which can be completely ruled out by current evidence. They include: (i) receptor-mediated intracellular signalling, (ii) influx/efflux of ions (other than Ca^2+^) through the *α*-LTX pore, and (iii) direct interactions with intracellular machinery by those parts of *α*-LTX that translocate into the cytosol.

Of these mechanisms, receptor-mediated signalling is perhaps the least likely cause of massive Ca^2+^-independent exocytosis. This is because the mutant LTX^N4C^, which stimulates neurotransmitter secretion via receptor signalling, seems to require extracellular Ca^2+^ for a strong action (Ashton et al., 2001; Capogna et al., 2003), although it is able to induce measurable exocytosis in the absence of Ca^2+^ (A. Zangrandi and Y. Ushkaryov, unpublished observations).

Both second and third proposed mechanisms require membrane insertion. In this respect, one should remember that all experimental paradigms used to assess Ca^2+^-independent *α*-LTX effects include Mg^2+^. Under these conditions, *α*-LTX will inevitably tetramerise (Orlova et al., 2000; Ashton et al., 2000; A. Rohou and Y. Ushkaryov, unpublished observation). Its insertion into the membrane would then lead to formation of tetrameric pores. Could it be that such pores activate exocytosis due to influx of ions, e.g. Mg^2+^? Support for this hypothesis comes from physiological experiments with the venom on frog NMJs (Misler and Hurlbut, 1979) and with *α*-LIT on blowfly NMJs (Magazanik et al., 1992). In these systems, the toxins stimulated strong asynchronous secretion in the absence of Ca^2+^, but removal of Mg^2+^ stopped this effect quickly and reversibly. In addition, calcium-independent effects are inhibited by La^3+^ and Cd^2+^ (Hurlbut et al., 1994; Scheer, 1989, 1990), and these cations are known to block the *α*-LTX pores (Sabirov et al., 1993; Chanturiya and Nikoloshina, 1994). Yet the ion permeability of the pore may not be sufficient to explain the effect of LTXs because hypertonic solutions can substitute for Mg^2+^ (Misler and Hurlbut, 1979).

The third hypothetical mechanism—direct interaction of parts of *α*-LTX with intracellular partners—is interesting. Some indirect evidence (Khvotchev and Sudhof, 2000; Li et al., 2005) and the presence of ARs, which are found only in intracellular proteins, have led these researchers to postulate that intracellular interactions of the toxin's N-terminus are involved in the Ca^2+^-independent effects of *α*-LTX. Although we would argue that the C-terminal *body* of *α*-LTX, rather than the N-terminus, can be implicated in this process (see Section 5.2 and Fig. 3C), this hypothesis still fails to explain the blockade of release by such extracellular factors as anti-toxin antibodies (Neri et al., 1965; Cattaneo and Grasso, 1986; Knipper et al., 1986), removal of Mg^2+^ (Misler and Hurlbut, 1979; Magazanik et al., 1992) and addition of La^3+^ (Scheer, 1989; Rosenthal et al., 1990). Thus, the Ca^2+^-independent mechanism of LTX action currently remains unexplained.

## 6 LITs as potential insecticides

When considering the use of LITs as potential insecticides, it is important to keep in mind that pore formation is by far their most toxic feature. Enhancing the ability to form pores, perhaps by creating covalently linked tetrameric molecules is one possibility. Lowering the specificity of toxin–receptor interaction (e.g. by mutagenesis or domain swapping) may help to target not only neuronal but also other insect tissues and in this way alleviate potential problems with variability of receptor expression.

On the other hand, depending on the localisation and functions of the insect orthologues(s) of LTX receptors, stimulation of the receptors may be an advantage, especially when one wants only to induce strong neurotransmitter secretion without the toxicity concomitant with pore formation. Alternatively, it may be possible to identify the minimal part of the LIT molecule that can still reproduce the effect of the toxin. Furthermore, it would be important to isolate and characterise endogenous ligands of LIT receptors. Such ligands may be further modified to increase their potency of receptor stimulation or they can be mimicked by chemical synthesis. Low molecular weight substitutes may also be easier to deliver.

Delivery of these large proteins can be based on the use of recombinant baculoviruses carrying the appropriate LIT genes. This approach has effectively been tested with *α*-LIT (Kiyatkin et al., 1995).

## References

[bib1] Adam-Vizi V., Deri Z., Bors P., Tretter L. (1993). Lack of involvement of [Ca^2+^]*_i_* in the external Ca^2+^-independent release of acetylcholine evoked by veratridine, ouabain and *α*-latrotoxin: possible role of [Na^+^]*_i_*. J. Physiol. Paris.

[bib2] Aicher B., Lerch M.M., Muller T., Schilling J., Ullrich A. (1997). Cellular redistribution of protein tyrosine phosphatases LAR and PTP*σ* by inducible proteolytic processing. J. Cell Biol..

[bib3] Andreev I., Danilevich V.N., Grishin E.V. (2005). Alternative splicing of pre-mRNA encoding the *Musca domestica* latrophilin-like protein (LLP): primary structures of four spliced forms of mRNA and their protein products. Bioorg. Khim..

[bib4] Ashton A.C., Rahman M.A., Volynski K.E., Manser C., Orlova E.V., Matsushita H., Davletov B.A., van Heel M., Grishin E.V., Ushkaryov Y.A. (2000). Tetramerisation of *α*-latrotoxin by divalent cations is responsible for toxin-induced non-vesicular release and contributes to the Ca^2+^-dependent vesicular exocytosis from synaptosomes. Biochimie.

[bib5] Ashton A.C., Volynski K.E., Lelianova V.G., Orlova E.V., Van Renterghem C., Canepari M., Seagar M., Ushkaryov Y.A. (2001). *α*-Latrotoxin, acting via two Ca^2+^-dependent pathways, triggers exocytosis of two pools of synaptic vesicles. J. Biol. Chem..

[bib6] Bettini S. (1971). On the mode of action of *Latrodectus* spp. venom. Ann. Ist. Super. Sanita..

[bib7] Biederer T., Sudhof T.C. (2000). Mints as adaptors. Direct binding to neurexins and recruitment of munc18. J. Biol. Chem..

[bib8] Boucard A.A., Chubykin A.A., Comoletti D., Taylor P., Sudhof T.C. (2005). A splice code for trans-synaptic cell adhesion mediated by binding of neuroligin 1 to *α*- and *β*-neurexins. Neuron %20.

[bib9] Broadie K., Prokop A., Bellen H.J., O’Kane C.J., Schulze K.L., Sweeney S.T. (1995). Syntaxin and synaptobrevin function downstream of vesicle docking in *Drosophila*. Neuron.

[bib10] Capogna M., Volynski K.E., Emptage N.J., Ushkaryov Y.A. (2003). The *α*-latrotoxin mutant LTX^N4C^ enhances spontaneous and evoked transmitter release in CA3 pyramidal neurons. J. Neurosci..

[bib11] Cattaneo A., Grasso A. (1986). A functional domain on the *α*-latrotoxin molecule, distinct from the binding site, involved in catecholamine secretion from PC12 cells: identification with monoclonal antibodies. Biochemistry.

[bib12] Cavalieri M., D’Urso D., Lassa A., Pierdominici E., Robello M., Grasso A. (1987). Characterization and some properties of the venom gland extract of a theridiid spider (*Steatoda paykulliana*) frequently mistaken for black widow spider (*Latrodectus tredecimguttatus*). Toxicon.

[bib13] Ceccarelli B., Hurlbut W.P. (1980). Ca^2+^-dependent recycling of synaptic vesicles at the frog neuromuscular junction. J. Cell Biol..

[bib14] Chang G.W., Stacey M., Kwakkenbos M.J., Hamann J., Gordon S., Lin H.H. (2003). Proteolytic cleavage of the EMR2 receptor requires both the extracellular stalk and the GPS motif. FEBS Lett..

[bib15] Chanturiya A.N., Nikoloshina H.V. (1994). Correlations between changes in membrane capacitance induced by changes in ionic environment and the conductance of channels incorporated into bilayer lipid membranes. J. Membr. Biol..

[bib16] Cull-Candy S.G., Neal H., Usherwood P.N. (1973). Action of black widow spider venom on an aminergic synapse. Nature.

[bib18] D’Ajello V., Mauro A., Bettini S. (1969). Effect of the venom of the black widow spider, *Latrodectus mactans tredecimguttatus*, on evoked action potentials in the isolated nerve cord of *Periplaneta americana*. Toxicon.

[bib17] D’Ajello V., Magni F., Bettini S. (1971). The effect of the venom of the black widow spider *Latrodectus mactans tredecimguttatus* on the giant neurones of *Periplaneta americana*. Toxicon.

[bib19] D’Amour F., Becker F.E., van Riper W. (1936). The black widow spider. Q. Rev. Biol..

[bib20] Danilevich V.N., Luk’ianov S.A., Grishin E.V. (1999). Cloning and structure of gene encoded *α*-latrocrustoxin from the Black widow spider venom. Bioorg. Khim..

[bib21] Davletov B.A., Krasnoperov V., Hata Y., Petrenko A.G., Sudhof T.C. (1995). High affinity binding of *α*-latrotoxin to recombinant neurexin I*α*. J. Biol. Chem..

[bib23] Davletov B.A., Shamotienko O.G., Lelianova V.G., Grishin E.V., Ushkaryov Y.A. (1996). Isolation and biochemical characterization of a Ca^2+^-independent *α*-latrotoxin-binding protein. J. Biol. Chem..

[bib22] Davletov B.A., Meunier F.A., Ashton A.C., Matsushita H., Hirst W.D., Lelianova V.G., Wilkin G.P., Dolly J.O., Ushkaryov Y.A. (1998). Vesicle exocytosis stimulated by *α*-latrotoxin is mediated by latrophilin and requires both external and stored Ca^2+^. EMBO J.

[bib24] Deri Z., Bors P., Adam-Vizi V. (1993). Effect of *α*-latrotoxin on acetylcholine release and intracellular Ca^2+^ concentration in synaptosomes: Na^+^-dependent and Na^+^-independent components. J. Neurochem..

[bib25] Dulubova I.E., Krasnoperov V.G., Khvotchev M.V., Pluzhnikov K.A., Volkova T.M., Grishin E.V., Vais H., Bell D.R., Usherwood P.N. (1996). Cloning and structure of *δ*-latroinsectotoxin, a novel insect-specific member of the latrotoxin family: functional expression requires C-terminal truncation. J. Biol. Chem..

[bib26] Finkelstein A., Rubin L.L., Tzeng M.C. (1976). Black widow spider venom: effect of purified toxin on lipid bilayer membranes. Science.

[bib27] Franklin C.E. (1988). Behavioural observations and neurophysiological responses of cockroaches envenomated with *Latrodectus katipo* venom. Comp. Biochem. Physiol. C..

[bib28] Fredriksson R., Gloriam D.E., Hoglund P.J., Lagerstrom M.C., Schioth H.B. (2003). There exist at least 30 human G-protein-coupled receptors with long Ser/Thr-rich N-termini. Biochem. Biophys. Res. Commun..

[bib29] Fritz L.C., Tzen M.C., Mauro A. (1980). Different components of black widow spider venom mediate transmitter release at vertebrate and lobster neuromuscular junctions. Nature.

[bib31] Frontali N., Grasso A. (1964). Separation of three toxicologically different protein components from the venom of the spider *Latrodectus tredecimguttatus*. Arch. Biochem. Biophys..

[bib30] Frontali N., Ceccarelli B., Gorio A., Mauro A., Siekevitz P., Tzeng M.C., Hurlbut W.P. (1976). Purification from black widow spider venom of a protein factor causing the depletion of synaptic vesicles at neuromuscular junctions. J. Cell Biol..

[bib32] Gasparini S., Kiyatkin N., Drevet P., Boulain J.C., Tacnet F., Ripoche P., Forest E., Grishin E., Menez A. (1994). The low molecular weight protein which co-purifies with *α*-latrotoxin is structurally related to crustacean hyperglycemic hormones. J. Biol. Chem..

[bib33] Graudins A., Padula M., Broady K.W., Nicholson G.M. (2001). Red-back spider (*Latrodectus hasselti*) antivenom prevents the toxicity of widow spider venoms. Ann. Emerg. Med..

[bib34] Graudins A., Gunja N., Broady K.W., Nicholson G.M. (2002). Clinical and in vitro evidence for the efficacy of Australian redback spider (*Latrodectus hasselti*) antivenom in the treatment of Brown cupboard spider (*Steatoda grossa*) envenomation. Toxicon.

[bib35] Gray J.X., Haino M., Roth M.J., Maguire J.E., Jensen P.N., Yarme A., Stetler-Stevenson M.A., Siebenlist U., Kelly K. (1996). CD97 is a processed, seven-transmembrane, heterodimeric receptor associated with inflammation. J. Immunol..

[bib36] Griffiths D.J., Smyth T. (1973). Action of black widow spider venom at insect neuromuscular junctions. Toxicon.

[bib37] Grishin E.V. (1998). Black widow spider toxins: the present and the future. Toxicon.

[bib38] Grishin E.V., Himmelreich N.H., Pluzhnikov K.A., Pozdnyakova N.G., Storchak L.G., Volkova T.M., Woll P.G. (1993). Modulation of functional activities of the neurotoxin from black widow spider venom. FEBS Lett..

[bib39] Harmar A.J. (2001). Family-B G-protein-coupled receptors. Genome Biol..

[bib40] Hata Y., Butz S., Sudhof T.C. (1996). CASK: a novel dlg/PSD95 homolog with an N-terminal calmodulin-dependent protein kinase domain identified by interaction with neurexins. J. Neurosci..

[bib41] Hayflick J.S. (2000). A family of heptahelical receptors with adhesion-like domains: a marriage between two super families. J. Recept. Signal Transduct. Res..

[bib42] Hein J. (1990). Unified approach to alignment and phylogenies. Methods Enzymol..

[bib43] Hlubek M.D., Stuenkel E.L., Krasnoperov V.G., Petrenko A.G., Holz R.W. (2000). Calcium-independent receptor for α-latrotoxin and neurexin 1α facilitate toxin-induced channel formation: evidence that channel formation results from tethering of toxin to membrane. Mol. Pharmacol..

[bib44] Hurlbut W.P., Chieregatti E., Valtorta F., Haimann C. (1994). *α*-Latrotoxin channels in neuroblastoma cells. J. Membr. Biol..

[bib46] Ichtchenko K., Khvotchev M., Kiyatkin N., Simpson L., Sugita S., Sudhof T.C. (1998). *α*-Latrotoxin action probed with recombinant toxin: receptors recruit *α*-latrotoxin but do not transduce an exocytotic signal. EMBO J..

[bib45] Ichtchenko K., Bittner M.A., Krasnoperov V., Little A.R., Chepurny O., Holz R.W., Petrenko A.G. (1999). A novel ubiquitously expressed *α*-latrotoxin receptor is a member of the CIRL family of G-protein-coupled receptors. J. Biol. Chem..

[bib47] Keegan H.L., Hedeen R.A., Whittemore F.W. (1960). Seasonal variation in venom of black widow spiders. Am. J. Trop. Med. Hyg..

[bib48] Khvotchev M., Sudhof T.C. (2000). *α*-Latrotoxin triggers transmitter release via direct insertion into the presynaptic plasma membrane. EMBO J..

[bib51] Kiyatkin N.I., Dulubova I.E., Chekhovskaya I.A., Grishin E.V. (1990). Cloning and structure of cDNA encoding *α*-latrotoxin from black widow spider venom. FEBS Lett..

[bib49] Kiyatkin N., Dulubova I., Chekhovskaya I., Lipkin A., Grishin E. (1992). Structure of the low molecular weight protein copurified with *α*-latrotoxin. Toxicon.

[bib50] Kiyatkin N., Dulubova I., Grishin E. (1993). Cloning and structural analysis of *α*-latroinsectotoxin cDNA. Abundance of ankyrin-like repeats. Eur. J. Biochem..

[bib52] Kiyatkin N.I., Kulikovskaya I.M., Grishin E.V., Beadle D.J., King L.A. (1995). Functional characterization of black widow spider neurotoxins synthesised in insect cells. Eur. J. Biochem..

[bib53] Knipper M., Madeddu L., Breer H., Meldolesi J. (1986). Black widow spider venom-induced release of neurotransmitters: mammalian synaptosomes are stimulated by a unique venom component (*α*-latrotoxin), insect synaptosomes by multiple components. Neuroscience.

[bib55] Krasilnikov O.V., Sabirov R.Z. (1992). Comparative analysis of latrotoxin channels of different conductance in planar lipid bilayers. Evidence for cluster organization. Biochim. Biophys. Acta.

[bib54] Krasil’nikov O.V., Ternovskii V.I., Sabirov R.Z., Tashmukhamedov B.A. (1985). Resistance of various protein channels to proteolytic degradation. Biofizika.

[bib56] Krasnoperov V., Lu Y., Buryanovsky L., Neubert T.A., Ichtchenko K., Petrenko A.G. (2002). Post-translational proteolytic processing of the calcium-independent receptor of *α*-latrotoxin (CIRL), a natural chimera of the cell adhesion protein and the G protein-coupled receptor. Role of the G protein-coupled receptor proteolysis site (GPS) motif. J. Biol. Chem..

[bib60] Krasnoperov V.G., Shamotienko O.G., Grishin E.V. (1990). Isolation and properties of insect-specific neurotoxins from venoms of the spider *Lactodectus mactans tredecimguttatus*. Bioorg. Khim..

[bib61] Krasnoperov V.G., Shamotienko O.G., Grishin E.V. (1991). Interaction of *α*-^125^latrocrustotoxin with nerve cell membranes from the river crab *Astacus astacus*. Bioorg. Khim..

[bib57] Krasnoperov V.G., Beavis R., Chepurny O.G., Little A.R., Plotnikov A.N., Petrenko A.G. (1996). The calcium-independent receptor of *α*-latrotoxin is not a neurexin. Biochem. Biophys. Res. Commun..

[bib58] Krasnoperov V.G., Bittner M.A., Beavis R., Kuang Y., Salnikow K.V., Chepurny O.G., Little A.R., Plotnikov A.N., Wu D., Holz R.W., Petrenko A.G. (1997). *α*-Latrotoxin stimulates exocytosis by the interaction with a neuronal G-protein-coupled receptor. Neuron.

[bib59] Krasnoperov V.G., Bittner M.A., Mo W., Buryanovsky L., Neubert T.A., Holz R.W., Ichtchenko K., Petrenko A.G. (2002). Protein tyrosine phosphatase-*σ* is a novel member of the functional family of *α*-latrotoxin receptors. J. Biol. Chem..

[bib62] Lelianova V.G., Davletov B.A., Sterling A., Rahman M.A., Grishin E.V., Totty N.F., Ushkaryov Y.A. (1997). *α*-Latrotoxin receptor, latrophilin, is a novel member of the secretin family of G protein-coupled receptors. J. Biol. Chem..

[bib63] Li G., Lee D., Wang L., Khvotchev M., Chiew S.K., Arunachalam L., Collins T., Feng Z.P., Sugita S. (2005). N-terminal insertion and C-terminal ankyrin-like repeats of *α*-latrotoxin are critical for Ca^2+^-dependent exocytosis. J. Neurosci..

[bib64] Longenecker H.E., Hurlbut W.P., Mauro A., Clark A.W. (1970). Effects of black widow spider venom on the frog neuromuscular junction. Effects on end-plate potential, miniature end-plate potential and nerve terminal spike. Nature.

[bib65] Loria P.M., Hodgkin J., Hobert O. (2004). A conserved postsynaptic transmembrane protein affecting neuromuscular signaling in *Caenorhabditis elegans*. J. Neurosci..

[bib66] Magazanik L.G., Fedorova I.M., Kovalevskaya G.I., Pashkov V.N., Bulgakov O.V., Grishin E.V. (1992). Selective presynaptic insectotoxin (*α*-latroinsectotoxin) isolated from black widow spider venom. Neuroscience.

[bib67] Majori G., Bettini S., Casaglia O. (1972). Effect of black widow spider venom on the cockroach heart. J. Insect. Physiol..

[bib68] Matsushita H., Lelianova V.G., Ushkaryov Y.A. (1999). The latrophilin family: multiply spliced G protein-coupled receptors with differential tissue distribution. FEBS Lett..

[bib69] McMahon H.T., Rosenthal L., Meldolesi J., Nicholls D.G. (1990). *α*-Latrotoxin releases both vesicular and cytoplasmic glutamate from isolated nerve terminals. J. Neurochem..

[bib70] Mee C.J., Tomlinson S.R., Perestenko P.V., De Pomerai D., Duce I.R., Usherwood P.N., Bell D.R. (2004). Latrophilin is required for toxicity of black widow spider venom in *Caenorhabditis elegans*. Biochem. J..

[bib71] Meir, A., 2003. *α*-Latrotoxin: a molecular tool for induction of neurotransmitter release. Alomone Labs Modulator No. 17.

[bib72] Meldolesi J., Madeddu L., Torda M., Gatti G., Niutta E. (1983). The effect of *α*-latrotoxin on the neurosecretory PC12 cell line: studies on toxin binding and stimulation of transmitter release. Neuroscience.

[bib73] Misler S., Hurlbut W.P. (1979). Action of black widow spider venom on quantized release of acetylcholine at the frog neuromuscular junction: dependence upon external Mg^2+^. Proc. Natl. Acad. Sci. USA.

[bib75] Missler M., Sudhof T.C. (1998). Neurexins: three genes and 1001 products. Trends Genet..

[bib74] Missler M., Fernandez-Chacon R., Sudhof T.C. (1998). The making of neurexins. J. Neurochem..

[bib76] Mosavi L.K., Cammett T.J., Desrosiers D.C., Peng Z.Y. (2004). The ankyrin repeat as molecular architecture for protein recognition. Protein Sci..

[bib77] Neri L., Bettini S., Frank M. (1965). The effect of *Latrodectus mactans tredecimguttatus* venom on the endogenous activity of *Periplaneta americana* nerve cord. Toxicon.

[bib78] Orlova E.V., Rahman M.A., Gowen B., Volynski K.E., Ashton A.C., Manser C., van Heel M., Ushkaryov Y.A. (2000). Structure of *α*-latrotoxin oligomers reveals that divalent cation-dependent tetramers form membrane pores. Nat. Struct. Biol..

[bib79] Ornberg R.L., Smyth T., Benton A.W. (1976). Isolation of a neurotoxin with A presynaptic action from the venom of the black widow spider (*Latrodectus mactans*, Fabr.). Toxicon.

[bib80] Pennuto M., Dunlap D., Contestabile A., Benfenati F., Valtorta F. (2002). Fluorescence resonance energy transfer detection of synaptophysin I and vesicle-associated membrane protein 2 interactions during exocytosis from single live synapses. Mol. Biol. Cell..

[bib81] Pescatori M., Bradbury A., Bouet F., Gargano N., Mastrogiacomo A., Grasso A. (1995). The cloning of a cDNA encoding a protein (latrodectin) which co-purifies with the *α*-latrotoxin from the black widow spider *Latrodectus tredecimguttatus* (Theridiidae). Eur. J. Biochem..

[bib82] Petrenko A.G., Kovalenko V.A., Shamotienko O.G., Surkova I.N., Tarasyuk T.A., Ushkaryov Yu.A., Grishin E.V. (1990). Isolation and properties of the *α*-latrotoxin receptor. EMBO J..

[bib83] Pot D.A., Woodford T.A., Remboutsika E., Haun R.S., Dixon J.E. (1991). Cloning, bacterial expression, purification, and characterization of the cytoplasmic domain of rat LAR, a receptor-like protein tyrosine phosphatase. J. Biol. Chem..

[bib84] Robbins N., Kuchynski M., Polak J., Grasso A. (1990). Motor nerve terminal restoration after focal destruction in young and old mice. Int. J. Dev. Neurosci..

[bib85] Rosenthal L., Meldolesi J. (1989). *α*-Latrotoxin and related toxins. Pharmacol. Ther..

[bib86] Rosenthal L., Zacchetti D., Madeddu L., Meldolesi J. (1990). Mode of action of *α*-latrotoxin: role of divalent cations in Ca^2+^-dependent and Ca^2+^-independent effects mediated by the toxin. Mol. Pharmacol..

[bib87] Sabirov R.Z., Iul’chibaeva N.A., Krasil’nikov O.V. (1993). Blocking of the latrotoxin channel by cadmium ions. Biofizika.

[bib88] Scheer H.W. (1989). Interactions between *α*-latrotoxin and trivalent cations in rat striatal synaptosomal preparations. J. Neurochem..

[bib89] Scheer H.W. (1990). Interactions between the presynaptically active neurotoxins *α*-latrotoxin and omega-conotoxin GVIA: studies on calcium fluxes and binding parameters in rat and chicken synaptosomes. Can. J. Physiol. Pharmacol..

[bib90] Scheiffele P., Fan J., Choih J., Fetter R., Serafini T. (2000). Neuroligin expressed in nonneuronal cells triggers presynaptic development in contacting axons. Cell.

[bib91] Sedgwick S.G., Smerdon S.J. (1999). The ankyrin repeat: a diversity of interactions on a common structural framework. Trends Biochem. Sci..

[bib92] Shatursky O.Y., Pashkov V.N., Bulgacov O.V., Grishin E.V. (1995). Interaction of *α*-latroinsectotoxin from *Latrodectus mactans* venom with bilayer lipid membranes. Biochim. Biophys. Acta.

[bib93] Smith D.S., Russell F.E., Russell F.E., Saunders P.R. (1966). Structure of the venom gland of the black widow spider *Latrodectus mactans*. A preliminary light and electron microscopic study. Animal Toxins.

[bib94] Snyder D.A., Rivers A.M., Yokoe H., Menco B.P., Anholt R.R. (1991). Olfactomedin: purification, characterization, and localization of a novel olfactory glycoprotein. Biochemistry.

[bib95] Stacey M., Lin H.H., Gordon S., McKnight A.J. (2000). LNB-TM7, a group of seven-transmembrane proteins related to family-B G-protein-coupled receptors. Trends Biochem. Sci..

[bib96] Sugita S., Khvochtev M., Südhof T.C. (1999). Neurexins are functional *α*-latrotoxin receptors. Neuron.

[bib97] Tabuchi K., Sudhof T.C. (2002). Structure and evolution of neurexin genes: insight into the mechanism of alternative splicing. Genomics.

[bib98] Tzeng M.C., Siekevitz P. (1978). The effect of the purified major protein factor (*α*-latrotoxin) of black widow spider venom on the release of acetylcholine and norepinephrine from mouse cerebral cortex slices. Brain Res..

[bib99] Umbach J.A., Grasso A., Zurcher S.D., Kornblum H.I., Mastrogiacomo A., Gundersen C.B. (1998). Electrical and optical monitoring of *α*-latrotoxin action at *Drosophila* neuromuscular junctions. Neuroscience.

[bib100] Ushkaryov Y. (2002). *α*-Latrotoxin: from structure to some functions. Toxicon.

[bib101] Ushkaryov Y.A., Petrenko A.G., Geppert M., Sudhof T.C. (1992). Neurexins: synaptic cell surface proteins related to the *α*-latrotoxin receptor and laminin. Science.

[bib102] Van Renterghem C., Iborra C., Martin-Moutot N., Lelianova V., Ushkaryov Y., Seagar M. (2000). *α*-Latrotoxin forms calcium-permeable membrane pores via interactions with latrophilin or neurexin. Eur. J. Neurosci..

[bib103] Verhage M., Maia A.S., Plomp J.J., Brussaard A.B., Heeroma J.H., Vermeer H., Toonen R.F., Hammer R.E., van den Berg T.K., Missler M., Geuze H.J., Sudhof T.C. (2000). Synaptic assembly of the brain in the absence of neurotransmitter secretion. Science.

[bib104] Volkova T.M., Pluzhnikov K.A., Woll P.G., Grishin E.V. (1995). Low molecular weight components from black widow spider venom. Toxicon.

[bib105] Volynski K.E., Capogna M., Ashton A.C., Thomson D., Orlova E.V., Manser C.F., Ribchester R.R., Ushkaryov Y.A. (2003). Mutant *α*-latrotoxin (LTX^N4C^) does not form pores and causes secretion by receptor stimulation. This action does not require neurexins. J. Biol. Chem..

[bib106] Volynski K.E., Silva J.-P., Lelianova V.G., Rahman M.A., Hopkins C., Ushkaryov Y.A. (2004). Latrophilin fragments behave as independent proteins that associate and signal on binding of LTX^N4C^. EMBO J..

[bib107] Volynski K.V., Meunier F.A., Lelianova V.G., Dudina E.E., Volkova T.M., Rahman M.A., Manser C., Grishin E.V., Dolly J.O., Ashley R.H., Ushkaryov Y.A. (2000). Latrophilin, neurexin and their signaling-deficient mutants facilitate *α*-latrotoxin insertion into membranes but are not involved in pore formation. J. Biol. Chem..

[bib108] Willson J., Amliwala K., Davis A., Cook A., Cuttle M.F., Kriek N., Hopper N.A., O’Connor V., Harder A., Walker R.J., Holden-Dye L. (2004). Latrotoxin receptor signaling engages the UNC-13-dependent vesicle-priming pathway in *C. elegans*. Curr. Biol..

